# Verses

**Published:** 1907-12

**Authors:** 


### VERSES.

### MY STYLE.

My style may be ole-fashioned,
  An' mebbe out er date,
But when I meet a friend o' mine,
  I stick ter thet ole trait
O' extendin' a hand wide open
  An' grasp thet o' yer friend,
Then jis tighten up a trifle—
  Thet makes yer friendship blend.

—Ohio Sun.

### NEW YEAR PROBABILITIES.

How vast the difference will be,
Between the mighty heap,
Of good resolves produced today,
And that their makers keep.

—Chas. E. Nichols, M. D.

### TIP, TIP, TIP.

He is home once more,
He has been away
To the gay seashore,
Where it's pay, pay, pay.
And the city's cliffs—walls gray and grim—
Are a source of unceasing joy to him.

He is home once more,
Back from his trip
To the gay seashore,
Where it's tip, tip, tip.
And the oozing pitch and the bubbling tar
Seem sweeter than ozone by far, by far!

—Washington (D. C.) Herald.

### THE NEGRO PLAYS NO MORE THE BANJO.

Wen music stirred him han an foot
Een slabry times long er go,
De Nigger played de fiddle sum,
But mosely hit wus banjo.

But now er days, sence freedum cum,
Wen e trow way pipe fer segar,
De fiddle dus hol secon place,
But banjo is giv up fer gittar.

No longer dus hit play de jig,
De cullud gent, hab hier tase
Ole banjo am dun gon ter res,
De gittar hab dun tuk hits place.

—B. H. Teague.

